# Axially Bound Magnetic Skyrmions: Glueing Topological Strings Across an Interface

**DOI:** 10.1021/acs.nanolett.2c00689

**Published:** 2022-04-22

**Authors:** Kejing Ran, Yizhou Liu, Haonan Jin, Yanyan Shangguan, Yao Guang, Jinsheng Wen, Guoqiang Yu, Gerrit van der Laan, Thorsten Hesjedal, Shilei Zhang

**Affiliations:** † School of Physical Science and Technology, ShanghaiTech University, Shanghai 200031, China; ‡ ShanghaiTech Laboratory for Topological Physics, ShanghaiTech University, Shanghai 200031, China; § RIKEN Center for Emergent Matter Science (CEMS), Wako 351-0198, Japan; ∥ National Laboratory of Solid State Microstructures and Department of Physics, Nanjing University, Nanjing 210093, China and Collaborative Innovation Center of Advanced Microstructures, Nanjing 210093, China; ⊥ Beijing National Laboratory for Condensed Matter Physics, Institute of Physics, Chinese Academy of Sciences, Beijing 100190, China; ∇ Diamond Light Source, Harwell Science and Innovation Campus, Didcot OX11 0DE, United Kingdom; ¶ Clarendon Laboratory, Department of Physics, University of Oxford, Parks Road, Oxford OX1 3PU, United Kingdom

**Keywords:** magnetic skyrmions, topological magnetism, resonant elastic X-ray scattering, 3D magnetic structures

## Abstract

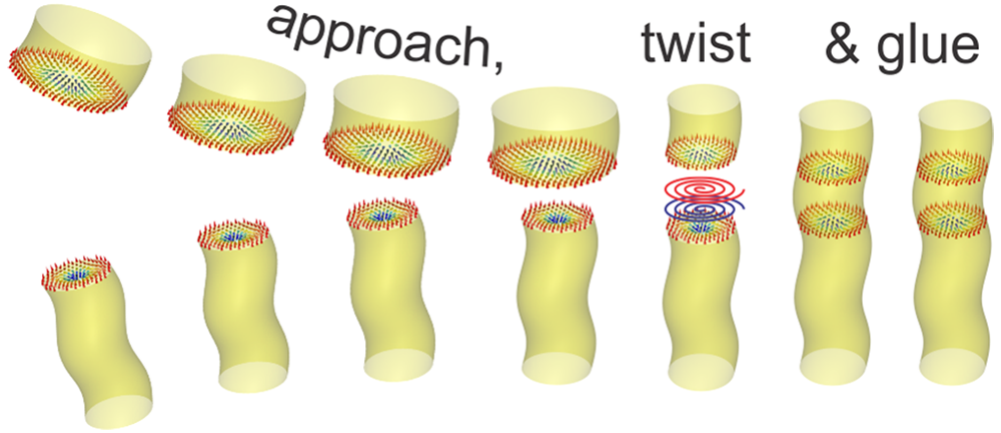

A major challenge in topological magnetism lies in the three-dimensional (3D) exploration of their magnetic textures. A recent focus has been the question of how 2D skyrmion sheets vertically stack to form distinct types of 3D topological strings. Being able to manipulate the vertical coupling should therefore provide a route to the engineering of topological states. Here, we present a new type of axially bound magnetic skyrmion string state in which the strings in two distinct materials are glued together across their interface. With quasi-tomographic resonant elastic X-ray scattering, the 3D skyrmion profiles before and after their binding across the interface were unambiguously determined and compared. Their attractive binding is accompanied by repulsive twisting; i.e., the coupled skyrmions mutually affect each other via a compensating twisting. This state exists in chiral magnet–magnetic thin film heterostructures, providing a new arena for the engineering of 3D topological phases.

All materials are composed of ensembles of particles in the presence of interactions. While attractive forces pull them together, the presence of repulsive forces is required for stable energy minima to occur, resulting in finite interparticle distances. In this Letter, we present an emergent version of this concept for topologically stabilized quasiparticles. We show that they can be vertically coupled together via an attractive interaction, while a repulsive interaction is also required to stabilize the ordered system.

Magnetic skyrmions are local solutions of the nonlinear two-dimensional (2D) soliton problem in magnetic systems,^1−9^ which induces emergent electromagnetism and extraordinary spin dynamics.^5,9^ The skyrmion–skyrmion interaction was subsequently discussed on the basis of a 2D model, leading to various ordering scenarios, mimicking the crystallization theory. It was therefore surprising to find the original 2D skyrmion crystal model being valid without considering their vertical, i.e., 3D, properties.

Although it was known that skyrmions extend into three dimensions by forming strings, reminiscent of superconducting vortex tubes,^10−13^ the question of how these 2D skyrmion sheets vertically stack to form 3D strings was not addressed. On the other hand, this question has become a central topic for topological magnetism, as even in a uniform, continuous material, skyrmion sheets can stack in a large variety of different ways^10,12^ and exhibit complex 3D excitation dynamics.^14^ The different ways of vertical stacking along *z* lead to distinct types of topological phases, featured by either a *d*_Sk_(*z*) or χ(*z*) profile, where *d*_Sk_ is the 2D skyrmion diameter and χ(*z*) is the helicity angle. The helicity angle is a measure of the tilting of the in-plane component of the magnetic moments and describes the type of 2D skyrmion (±90°, left- and right-handed Bloch-type skyrmions; 0° and 180°, divergent and convergent Néel-type skyrmions).

Taking confined chiral magnets as an example, the breaking of translational symmetry at the terminating surfaces leads to surface instabilities. Consequently, the 2D skyrmion sheets are no longer uniformly aligned near the surface but exhibit *surface twisting* with an exponential χ(*z*) profile instead.^15^ On the other hand, in multilayered thin film systems that are inherently discontinuous along the depth, dipolar interactions between the layers encourage a different χ(*z*) twisting profile (*dipolar twist*), suggesting a different vertical interaction scheme.^16,17^ Moreover, a modified energy landscape leads to a varying skyrmion profile as a function of depth (*d*_Sk_(*z*)), establishing new types of topological order, such as chiral bobbers^18−22^ and torons.^23^ Despite recent advances in the experimental exploration of the 3D nature of magnetic skyrmions,^22,24−30^ a deeper understanding of the “vertical” interactions in 3D structures on a microscopic level is required before effective models of skyrmion quasiparticle interactions, e.g., in the context of the Thiele equation,^31^ can be established.^32^

From an experimental perspective, a promising strategy is to design a materials system in which dissimilar skyrmion phases can be joined together via the tuning of the experimental measurement parameters. The observation of the coupling process could offer an unprecedented clear view of the vertical binding mechanism for topological objects. Here, we achieved the controlled merging of two distinct skyrmion species across a materials interface in a chiral magnet-multilayer heterostructure and were able to gain insight on how skyrmions behave across the interface. Our findings illustrate how the classical perception of the formation of ordered materials via the balance of attractive and repulsive forces is also applicable to the understanding of the vertical binding of topological objects.

## Results and Discussion

**Figure 1:**
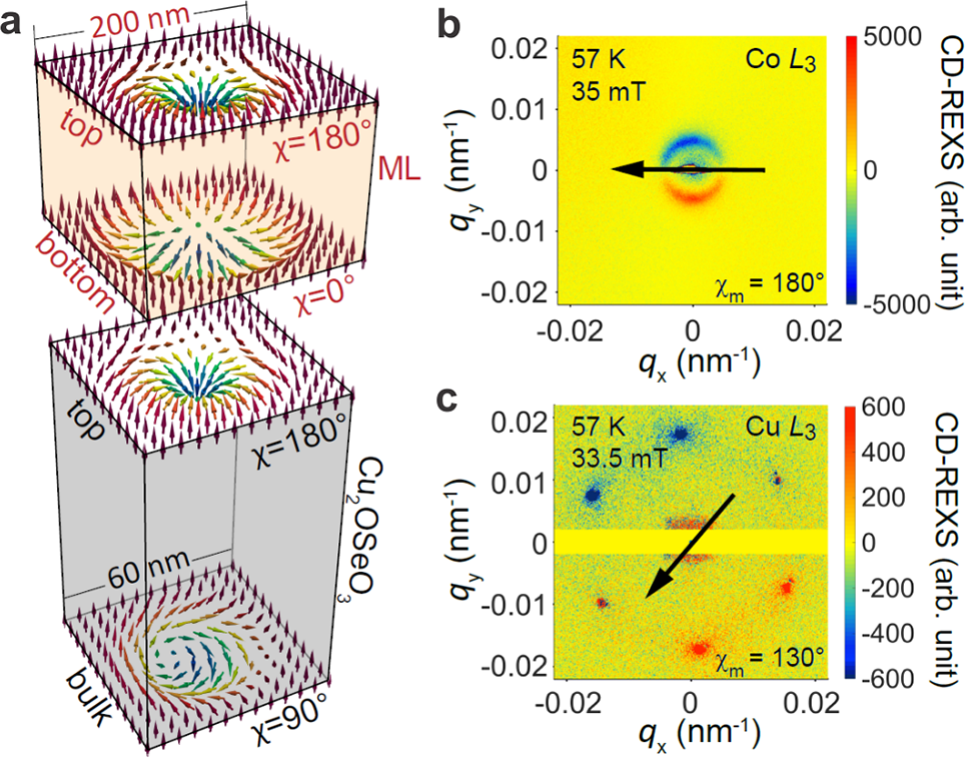
Skyrmion states in the uncoupled, isolated materials. (a) Depth dependence of the helicity angle χ in both the multilayer (ML) (above), reaching from 180° at the top to 0° at the bottom, and the pristine Cu_2_OSeO_3_ crystal (below), reaching from 180° at the surface to 90° in the bulk. CD-REXS patterns measured in the skyrmion phase for (b) a Si/ML at the Co L_3_ edge and (c) pristine Cu_2_OSeO_3_ at the Cu L_3_ edge.

For being able to control and observe the vertical stacking of 2D skyrmions, we conceived a model system centered around an interface between two 3D skyrmion configurations, namely, a dipolar twisted skyrmion string in a multilayer (ML) system and surface-twisted skyrmion string in a chiral magnet (which exists at any terminating surface). In this work, we separate the two skyrmion-harboring materials by a 3 nm thick Ta spacer, preventing their direct exchange coupling. This simplifies the system greatly in that the dipolar interaction is the only vertically binding energy term that has to be considered here. Figure 1a shows the relaxed 3D magnetic structures obtained from micromagnetic simulations^33^ for a ML (upper panel) and the chiral magnet Cu_2_OSeO_3_ (bottom panel), for the case of uncoupled, isolated systems (Supplementary Section S4). The Ta/[CoFeB/MgO/Ta]_4_ ML has a characteristic planar correlation length of λ_h_ ≈ 200 nm,^17^ which can be used as an estimate for the skyrmion size *d*_Sk_ in the ML. Most importantly, the presence of interfacial Dzyaloshinskii–Moriya interaction (DMI) and interlayer dipolar coupling results in a dipolar twist, exhibiting a hybrid 3D skyrmion string.^16,17,34^ As shown in Figure 1a, the helicity angle undergoes a π-turn from χ = 180° at the top to χ = 0° at the bottom. Note that both the top and bottom surfaces exhibit Néel-type skyrmions.

The other skyrmion string was chosen to be a surface-twist type system, which occurs, e.g., in Cu_2_OSeO_3_ single crystals with λ_h_ ≈ 60 nm^35,36^ (Figure 1a, below). The surface-twisted 3D skyrmion string is characterized by an exponential decay of its χ(*z*) profile:^15,25,26,37^
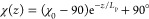
1where χ_0_ describes the helicity angle of the very top surface and *L*_p_ is the length scale characterizing the penetration depth of the surface twisting. Recently, experimental studies uncovered a pronounced, deep-reaching surface twist effect in Cu_2_OSeO_3_ (i.e., χ_0_ is 180°, while *L*_p_ measures ∼50 nm^25,26^), indicating the emergence of DMI at the surface level. We have included this additional DMI term in the simulations (Supplementary Section S4), which are able to reproduce the observed enhanced surface twist phenomenon. As shown in Figure 1a, the top surface becomes purely Néel-type, whereas in the bulk, χ returns to 90° which is the expected value for Bloch-type skyrmions.^25^

We first examine the individual skyrmion string structures in the two materials separately. Pristine Cu_2_OSeO_3_ single crystals and pristine MLs were synthesized (Supplementary Section S1), and their chiral and topological properties were characterized by circular dichroism in resonant elastic X-ray scattering (CD-REXS, Supplementary Sections S2 and S3).^16,17,25,26,38−41^ Such element-specific experiments were performed using synchrotron-generated soft X-rays, tuned to the *L*_2,3_ absorption edges of the 3d magnetic ions,^40^ at the RASOR diffractometer on beamline I10 at the Diamond Light Source (Oxfordshire, UK). Here, by tuning the photon energy to the Co and Cu L-edges (Co, 774–800 eV; Cu, 925–960 eV), respectively, CD-REXS is able to selectively target the 3D skyrmion string’s internal structure in either the ML or the chiral magnet.

The scattering results are best presented as a reciprocal space map in the *q*_*x*_-*q*_*y*_ plane at *q*_*z*_ = 0.^36^ For a 2D skyrmion plane, the CD amplitude *I* as a function of azimuthal angle Ψ follows *I*(Ψ) = *Y* sin(Ψ + χ),^26,40^ where *Y* is a constant for a particular scattering configuration and photon energy. In other words, the rotation angle of the extinction vector uniquely reveals the χ value, as illustrated by the look-up tables in the Supplementary Figures S4 and S5. For three-dimensional systems with nonuniform χ(*z*), the measured CD-REXS intensity, *I*_m_, is thus averaged over all depths with a particular weighing factor *b*(*z*), which is depth-dependent.^22,42^*I*_m_ can be written as
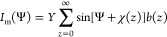
2where *b*(*z*) = e^–2*z* sec^ ^α/Λ(*ℏω*)^/Λ(*ℏω*), Λ is the X-ray penetration length which is a function of photon energy *ℏω*, and α is the incident angle with respect to the surface normal. It can be seen from eq 2 that *I*_m_(Ψ) also takes a sinusoidal profile with a half-positive–half-negative CD pattern, from which one can identify a distinct extinction vector, and an associated χ_m_ value.^26^ It is thus worth emphasizing that the experimental value χ_m_ represents an average over the specific χ(*z*) configuration.

Figure 1b shows the CD-REXS pattern measured on the ML sample, using nonmagnetic Si as a substrate, for skyrmion-stabilizing conditions of 57 K and 35 mT, and with the energy tuned to the Co L_3_ edge.^17^ First, a ring-like pattern is observed, suggesting that skyrmions are rather disordered within the ML, forming a “polycrystalline” arrangement.^17^ Second, the observed *q*_h_ = 0.0047 nm^–1^ corresponds to a modulation periodicity of 213 nm. Note that λ_h_ is not an accurate measure of *d*_Sk_ in ML systems as the skyrmion size is field-dependent. Third, the CD-REXS pattern shows the characteristic half-positive–half-negative contrast, and from the orientation of the extinction vector (black arrow in Figure 1b), χ_m_ is found to be 180°. Taking depth averaging of χ(*z*) into account,^17,26^ the measured χ_m_ helicity angle is consistent with the dipolar twist model (Figure 1a).^17^

On the other hand, Figure 1c shows CD-REXS data in the skyrmion lattice phase on a pristine, (001)-oriented Cu_2_OSeO_3_ substrate, measured at 57 K and 33.5 mT. First, the 6-fold-symmetric diffraction pattern identifies a long-range ordered, hexagonal skyrmion lattice.^35,36^ Second, the diffraction wavevector *q*_*h*_ = 1/λ_*h*_ measures 0.0172 nm^–1^, yielding a skyrmion lattice constant of ∼66 nm,^35^ which is approximately the intrinsic skyrmion diameter in this compound. Third, from the orientation of the extinction vector in Figure 1c, χ_m_ = 130° is found. This value is also consistent with the surface twist model with an exponentially decaying χ(*z*) profile, described by eq 1 with χ_0_ = 180° and *L*_p_ = 48 nm, in agreement with previous reports.^25^

**Figure 2:**
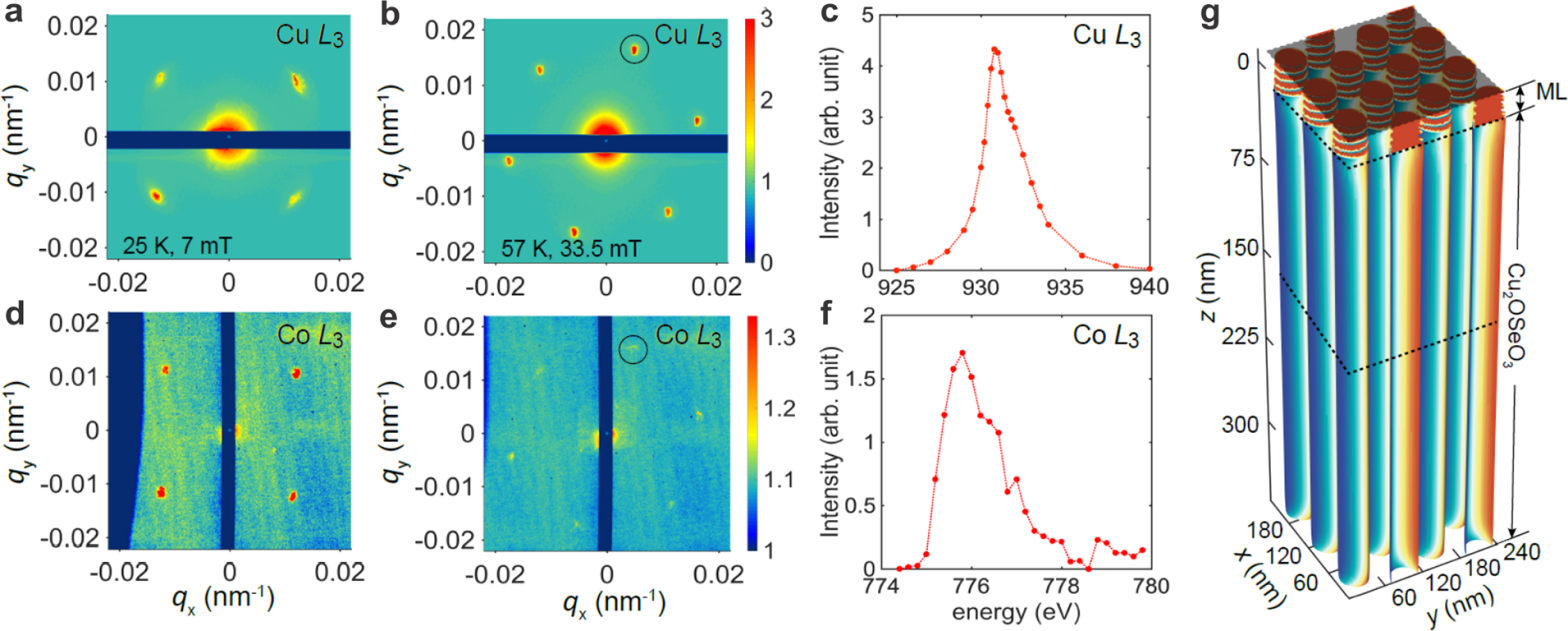
REXS patterns for the helical and skyrmion phase in the coupled Cu_2_OSeO_3_/ML skyrmion system. Tuning the photon energy to the Cu L_3_ edge, (a) four helical and (b) six skyrmion diffraction peaks are selectively measured in the Cu_2_OSeO_3_ bulk crystal. Note that REXS patterns are obtained by mapping out the respective (curved) section of reciprocal space, followed by the calculation of the scattering patterns in, e.g., the *q*_*x*_–*q*_*y*_ plane at *q*_*z*_ = 0, as shown in the figures. (c) Photon energy spectrum of the magnetic peak (circled in (b)) intensity near the Cu L_3_ edge (from 929 to 933 eV). Tuning the photon energy to the Co L_3_ edge, the selective diffraction off the ML also reveals (d) four helical and (e) six skyrmion peaks, demonstrating the imprinting effect. (f) Energy spectrum of the magnetic peak (circled in (e)) intensity near the Co L_3_ edge (from 774 to 780 eV). (g) 3D magnetic structure for a coupled chiral bulk-ML magnet system, obtained from micromagnetic simulations.

Next, the ML and the chiral bulk magnet are joined together, forming a single heterostructure; i.e., the Cu_2_OSeO_3_ single crystal serves as a substrate for the subsequent thin film heterostructure growth, following a careful surface treatment (Supplementary Section S1). It is worth noting that the identical Cu_2_OSeO_3_ substrate (Figure 1c) was used for the REXS observations before and after the heterostructure was synthesized, thereby providing a reliable control for the observation of vertical skyrmion stacking. Figure 2 panels a and b show the standard REXS patterns for the heterostructure sample at the Cu L_3_ edge, and specifically the characteristic magnetic structures in Cu_2_OSeO_3_ in the near-interface region. The four-spot pattern measured at 25 K and 7 mT (Figure 2a) suggests helical order, while the 6-fold-symmetric pattern at 57 K and 33.5 mT (Figure 2b) reveals the formation of the skyrmion string lattice. In both states, the real-space periodicity is the same as that measured on the pristine Cu_2_OSeO_3_ substrate (Figure 1c), indicating that the skyrmion lattice constant, as well as the skyrmion size, remain unchanged in the bulk crystal after coupling to the ML. Furthermore, the photon energy-dependent measurement at a fixed skyrmion lattice wavevector (Figure 2c) confirms that the measured signals are indeed solely from Cu_2_OSeO_3_.

We then probe the magnetic structures in the ML for the same measurement parameters (temperatures and fields) used for obtaining the Cu_2_OSeO_3_ data shown in Figures 2a,b, however, with the photon energy tuned to the Co L_3_ edge. First, in Figure 2d, the REXS result from the ML in the helical state of Cu_2_OSeO_3_ is shown. Strikingly, the ring-shaped scattering pattern characteristic for the uncoupled ML (Figure 1b) has drastically changed and now exhibits four well-defined diffraction spots. The *q*_*h*_ is identical to that of uncoupled Cu_2_OSeO_3_ (Figure 2a); i.e., *q*_*h*_ in the ML increases by more than 3 times. A straightforward interpretation is that the magnetic coupling across the interface “imprints” the helical order from the Cu_2_OSeO_3_ onto the ML. Consequently, the periodicity of the initial stripe domains in the ML shrink from ∼200 to ∼60 nm and are now locked along particular azimuthal directions, as governed by the anisotropy of the chiral magnet. Second, in the skyrmion lattice phase of Cu_2_OSeO_3_ (Figure 2e), the ML shows a 6-fold-symmetric magnetic diffraction pattern identical to that of the uncoupled Cu_2_OSeO_3_ bulk crystal (Figure 2b), however, with reduced scattering intensity. This 6-fold pattern is characteristic for a hexagonal packing of the skyrmion lattice. The reduced intensity can, in principle, have two origins. First, as the interaction between the two 3D skyrmion strings is primarily due to the dipolar interaction, the ML layer closest to the interface encounters a stronger attraction from the chiral magnet than the layers further away from the interface. This may result in partial vertical binding; i.e., the imprinting effect from the Cu_2_OSeO_3_ is gradually decreasing with distance from the interface. Second, due to the large difference in their undisturbed skyrmion sizes, perfect vertical stacking may not occur over a large lateral area, but instead only across limited-size domains. Note, however, that both effects should result in a larger diffuse scattering background, which is not observed in Figure 2e.

The energy scan for fixed *q*_h_ (Figure 2f) further supports the finding that the measured magnetically ordered patterns (Figure 2d,e) are indeed from the ML. By comparing with the uncoupled case (Figure 1b), it is clear that by stacking two independent skyrmion strings on top of each other (across an interface), the bottom surface of the dipolar-twisted skyrmion adapts a size compatible with the one of the chiral magnet. Moreover, the two skyrmion string species are locally glued together, forming a single, continuous 3D string. This attractive feature is accompanied by a shrinking of the size of the skyrmions, which subsequently assemble into a long-range ordered lattices—a phenomenon not commonly observed in MLs. Next, we carried out micromagnetic simulations of the vertical binding phenomenon (Figure 2g; Supplementary Section S4). The simulation results were obtained using realistic materials parameters for the ML,^17^ and they reveal attractive type vertical binding across the interface, in agreement with our REXS observations.

**Figure 3:**
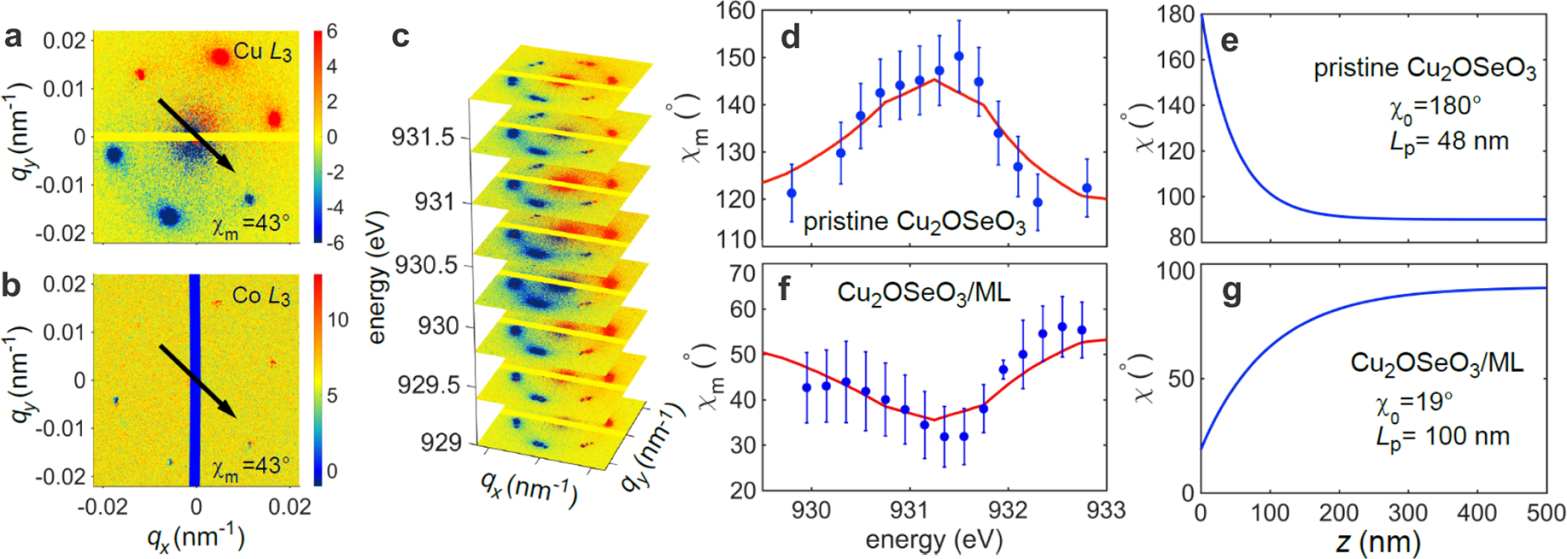
Depth-resolved CD-REXS of the Cu_2_OSeO_3_/ML heterostructure sample. CD-REXS patterns measured in the skyrmion lattice phase of Cu_2_OSeO_3_ at the (a) Cu and (b) Co L_3_ edges. For the purpose of this work, we define the circular dichroism signal (the CD-REXS signal) as the difference in diffraction intensity for the same skyrmion peak at the same geometrical condition, obtained using left- and right-circularly polarized soft X-rays. (c) CD-REXS pattern for different incident photon energies across the Cu L_3_ absorption edge. Note that by using CD-REXS, the helicity angle χ of a 2D skyrmion can be unambiguously determined.^17,25,26,39^ (d) For comparison, χ_*m*_ was measured as a function of photon energy for the pristine Cu_2_OSeO_3_ substrate. The red curve represents the best fit to the experimental data points (blue dots), using the depth dependence of χ(*z*) shown in (e). (f,g) χ_*m*_(*ℏω*) and χ(*z*) for the Cu_2_OSeO_3_/ML heterostructure sample. Note that the surface twist penetration depth *L*_p_ is different for the two samples.

In order to fully analyze the detailed 3D χ(*z*) profile of the bound skyrmion string, a systematic CD-REXS study was performed. First, the vertically averaged helicity angle χ_m_ of the bound state was measured. Figure 3 panels a and b show the CD-REXS patterns and extinction vectors for the heterostructure sample, probing Cu_2_OSeO_3_ (at the Cu L_3_ edge) and the ML (at the Co L_3_ edge), respectively. Surprisingly, as shown in Figure 3a, after binding the two skyrmion species together, χ_m_ undergoes a fundamental change from 130° (Figure 1c) to 43° at the near-surface region of the chiral magnet. This immediately suggests that the average skyrmion type drastically changes from a convergent swirl to a divergent vortex (look-up tables in Supplementary Figures S4 and S5). At the same time (Figure 3b), the average helicity angle of the ML also takes the value of 43°, indicating that the two contacting 2D skyrmion sheets are seamlessly fused together.

Next, the 3D χ(*z*) profile of the bound state is quantitatively measured using depth-dependent CD-REXS. Equation 2 suggests that by varying the photon energy *ℏω*, *b*(*z*) can be adjusted, leading to a different χ_m_ that effectively probes a different volume of the sample.^25^ By systematically probing χ_m_(*ℏω*), the actual depth dependence χ(*z*) can be reconstructed, analogous to the concept of tomography. Figure 3c shows the *I*_m_ pattern obtained at different photon energies across the Cu L_3_ edge for the heterostructure sample. The amplitude of the CD signal is governed by *Y*,^40^ which varies with the cross-section of the X-ray absorption, therefore being energy-dependent across the L_3_ edge. Nevertheless, this does not affect the accuracy of the measured χ_m_, as it only depends on the rotation angle of the extinction vector.^26^ It is thus clear that the extinction vector undergoes a gradual rotation upon varying the photon energy, indicative of the underlying 3D χ(*z*) profile.

Figure 3d shows the measured χ_m_ as a function of *ℏω* (blue dots) for the pristine Cu_2_OSeO_3_ substrate. The measured data are analyzed using a self-consistent fitting algorithm by assuming a 3D χ(*z*) structure, inserting it into eq 2, and iteratively optimizing χ(*z*) until a good agreement between model and data is reached. Figure 3e shows the best-fit model for χ(*z*) that is used to produce the red curve in Figure 3d. As expected, the pristine Cu_2_OSeO_3_ crystal shows the surface twist effect with χ_0_ = 180° convergent skyrmions on the very top surface, undergoing an exponential decay (*L*_p_ = 48 nm) while approaching to the bulk value of 90° (Bloch-type skyrmion).

**Figure 4:**
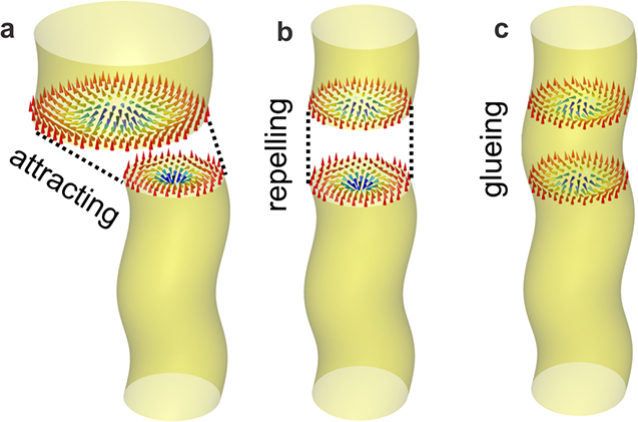
Glueing of skyrmion strings across an interface. (a) Sketch of two unperturbed skyrmion strings as they are characteristic for their respective host materials. Resulting from the dipolar coupling, the cores of the skyrmions attract each other. Due to their topological nature, skyrmions can deform without “breaking” (i.e., maintaining their winding number), adapting their size and shape to a changing environment. (b) Therefore, the bottom skyrmion surface in the ML shrinks and is glued onto the top skyrmion surface of the chiral magnet. (b) However, in between, where the moments rotate differently in skyrmion systems with different helicity angles, the coupling is repulsive with the rotation of the two systems mutually canceling each other to some degree. Note that the skyrmion size in the Cu_2_OSeO_3_ bulk crystal is not noticeably changing due to its larger overall stray field. The connection of a divergent and a convergent vortex will result in a repulsive force due to their opposite internal structures. The consequence of this repulsion is the “neutralization” effect in their helicity angles; i.e., both 3D skyrmion strings have to modify their χ(*z*) profiles to make the connection work. (c) Finally, glued 3D string structures are stabilized near the interface with adaptable size and compatible helicity angles, forming a continuous skyrmion string.

Figure 3 panels f and g show the measured χ_m_ and the analyzed χ(*z*) profile for the Cu_2_OSeO_3_/ML heterostructure sample, respectively. A remarkable feature is that the detailed depth-dependent twisting behavior is very different from that of the pristine Cu_2_OSeO_3_ crystal (Figure 3d,e). First, the top surface vortex orientation has been turned around (χ_0_ = 19°), and χ gradually recovers to standard Bloch-type skyrmion behavior toward the bulk. Second, the helicity modification effect reaches *L*_p_ ≈ 100 nm, much deeper than the intrinsic surface twist effect^15,25,26,37^ and also twice as deep as the helicity angle twist in pristine Cu_2_OSeO_3_. It is thus clear that near the interface, the intrinsically convergent skyrmion swirls in the surface region of Cu_2_OSeO_3_ become oppositely rotated, while the intrinsically divergent skyrmion swirl at the ML bottom surface is also turned in the opposite orientation, resulting in an intermediate helicity for both. Unfortunately, due to the suppressed CD-REXS signal from the ML, the reconstruction of the 3D χ(*z*) profile was not clear enough to be accessible. Nevertheless, it can be concluded that although an attractive force locks the two distinct skyrmions in position, there exists a more subtle repelling force due to the opposite helicity rotation, leading to significantly modified skyrmion helicity angles in both skyrmion systems near the interface. The vertical bindinging process for skyrmions is illustrated in Figure 4.

In summary, we have unambiguously observed the glueing of two distinct types of skyrmion strings. The vertical binding of the two skyrmion surfaces is a combination of attractive and repulsive interactions, akin to the general concept particles assembling into ordered structures. Although the observed coupling across the interface is relying on the dipole–dipole interaction, the skyrmion glueing process will also be observable for systems coupled via the direct exchange interaction as the primary terms in their Hamiltonians share strong similarities.^43^ An example of the latter are the uniform 3D tube structures found in chiral bulk crystals, which are composed of straightly stacked 2D skyrmions.^44^ Our findings unravel the microscopic details of topological 3D quasiparticle interaction, and they also demonstrate a new route toward creating and engineering new types of 3D topological phases, as the skyrmion glueing concept can be generally applied to a wide variety of materials species. It is intriguing to note that during the glueing process, the disordered, large-diameter ML skyrmions are compressed down to the dimensions of chiral magnet skyrmions, while also inheriting their lattice order. Further, the modification of the χ(*z*) skyrmion twist will change how an applied torque will drive the bound skyrmion system, resulting in different current–velocity relationships and skyrmion Hall angles.^32,45^ From this perspective, the glueing effect offers an effective strategy for the manipulation of the dynamic skyrmion properties, enabling applications.

## Supporting Information Available

Section S1, sample preparation; section S2, resonant elastic X-ray scattering; section S3, look-up table for determination of the helicity angle; section S4, micromagnetic simulations; Figure S1, atomic force microscopy image of the polished Cu_2_OSeO_3_ surface; Figure S2, REXS probing geometry; Figure S3, CD-REXS look-up table for a system with positive polarity; Figure S4, the same for a system with negative polarity (PDF)

## Notes

The data that support the findings of this study are available from the corresponding author on request.
