# Supplementary material for: Axially Bound Magnetic Skyrmions: Glueing Topological Strings Across an Interface
Source: Nano Lett. 2022 Apr 22;22(9):3737–43. doi: 10.1021/acs.nanolett.2c00689 (PMC9101076; doi:10.1021/acs.nanolett.2c00689)
Supplement: Supplementary file 1 — nl2c00689_si_001.pdf [file nl2c00689_si_001.pdf]

— Supplemental Material —

Axially Bound Magnetic Skyrmions: Glueing Topological Strings Across an Interface

Kejing Ran[†,1] Yizhou Liu[†,2] Haonan Jin,[1] Yanyan Shangguan,[3] Yao Guang,[4] Jinsheng Wen,[3] Guoqiang Yu,[4] Gerrit van der Laan,[5] Thorsten Hesjedal[*,6] and Shilei Zhang[*1]

[1]*School of Physical Science and Technology, ShanghaiTech University, Shanghai 200031, China and ShanghaiTech Laboratory for Topological Physics, ShanghaiTech University, Shanghai 200031, China*

[2]*RIKEN Center for Emergent Matter Science (CEMS), Wako 351-0198, Japan*

[3]*National Laboratory of Solid State Microstructures and Department of Physics, Nanjing University, Nanjing 210093, China and Collaborative Innovation Center of Advanced Microstructures, Nanjing 210093, China*

[4]*Beijing National Laboratory for Condensed Matter Physics, Institute of Physics, Chinese Academy of Sciences, Beijing 100190, China*

[5]*Diamond Light Source, Harwell Science and Innovation Campus, Didcot, OX11 0DE, United Kingdom*

[6]*Clarendon Laboratory, Department of Physics, University of Oxford, Parks Road, Oxford, OX1 3PU, United Kingdom*

Here, we first introduce the sample preparation details, followed by a description of resonant elastic x-ray scattering measurements, providing details of the experimental setup, the data acquisition, and the data processing. Next, we present look-up tables for determining the skyrmion helicity angles from a CD-REXS experiment. Finally, micromagnetic simulation details are provided.

S1. SAMPLE PREPARATION

Single crystals of Cu_2OSeO_3 were grown using the chemical-vapor-transport method. First, polycrystalline Cu_2OSeO_3 was synthesized by a solid-state reaction using a 2:1 molar ratio of CuO (Ventron, 99.999%) and SeO_2 (Alfa Aesar, 99.4%). The reaction mixture was sealed in an evacuated fused silica ampule and ramped to 300°C with a dwell time of 2 days and then raised to 600°C maintained for 7 days. The resulting polycrystalline material was ground, mixed together with NH_4Cl (i.e., the transport agent), and again sealed in an evacuated fused silica ampule. The source and the sink zones of the 2-zone furnace were ramped from 50 to 650 and 540°C, respectively. This temperature gradient was kept for 6 weeks. Finally, the furnace was ramped to RT within 5 h. Single crystals with shiny facets were collected from the deposition zone of the ampule and subsequently characterized by single crystal diffraction (using Cu $K\alpha$ radiation), confirming the high crystalline quality and single-chirality.

Subsequently, the Cu_2OSeO_3 (001) surface was oriented and prepared using a combination of lapping and polishing techniques. The surface quality of the Cu_2OSeO_3 (001) substrates were checked using atomic force microscopy (AFM) before depositing the thin film multilayers (see Fig. S1). The root mean square roughness of the as-polished surface is ∼1.5 nm (measured over a $20 \times 20\,\mu m^2$ region), confirming a wafer-level surface flatness.

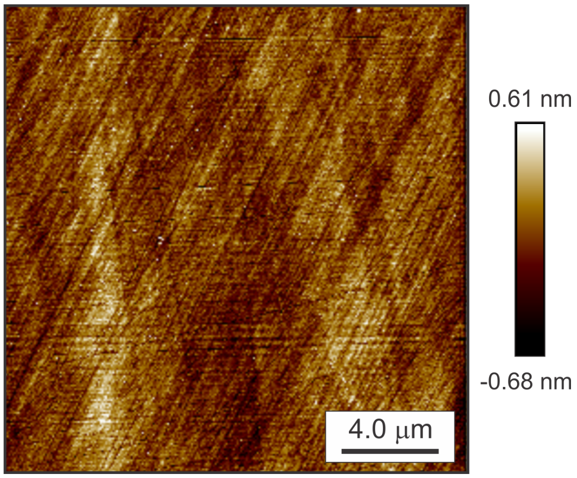

FIG. S1. AFM image of the polished Cu_2OSeO_3 crystal.

Next, $[Ta(2)/CoFeB(1.5)/MgO(2)]_4$ (thicknesses in nm) multilayer thin films were grown by magnetron sputtering, after annealing the as-polished Cu_2OSeO_3 substrate at 400°C for 2 h. Ar was used as the sputter gas at a pressure of 0.3 Pa. The base pressure of the deposition system was $<3\times10^{-6}$ Pa. All multilayers were capped with Ta to prevent oxidation. The deposition rates for Ta, CoFeB, and MgO were 0.48, 0.30, and 0.05 Å/s, respectively, using a power of 100 W.

Note that the Ta layer, separating the ML from the Cu_2OSeO_3 substrate, has been chosen thick enough to prevent the direct exchange coupling. This Ta layer has no effect on the magnetic state of the Cu_2OSeO_3 bulk

crystal, as shown in the Supplementary Material to Ref. [1].

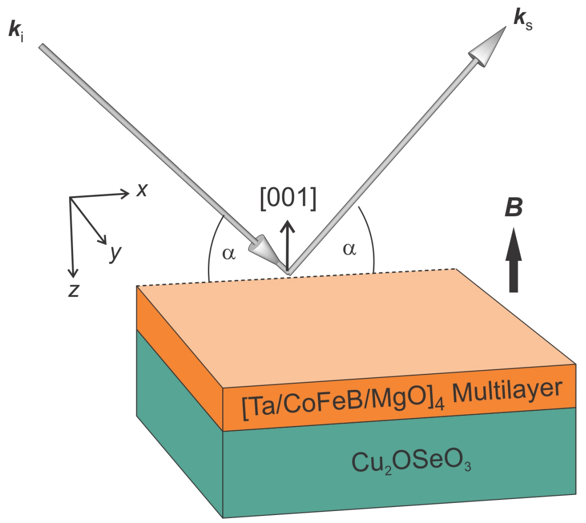

FIG. S2. Experimental CD-REXS geometry and coordinate system used in this work.

S2. RESONANT ELASTIC X-RAY SCATTERING

Resonant elastic x-ray scattering (REXS) experiments were carried out in the RASOR diffractometer on beamline I10 at the Diamond Light Source (UK). The scattering geometry and the coordinate system used for the successive calculation are illustrated in Fig. S2. The [001] surface normal direction of Cu_2OSeO_3 is indicated. The magnetic field is applied along the $-z$-direction. The incident and scattered x-ray wavevectors are denoted as $\mathbf{k}_i$ and $\mathbf{k}_s$, respectively, i.e., the scattering plane is the x-z plane. It is worth emphasizing that the incident angle α is not equal to the scattering angle of $\frac{1}{2}(2\theta)$, as the former relates to the x-ray probing depth, while the latter relates to the scattering wavevector. In this work, α varies from 3°-75° in order to cope with a wide range of photon energies covering a range from 700 to 960 eV.

We first performed standard x-ray absorption spectroscopy (XAS) measurements to identify the absorption edges of Co and Cu. Next, scattering experiments were carried out using an area detector (pixel size = 13.5 μm) which was placed 130 mm away from the diffraction center. REXS patterns were obtained by firstly mapping out a curved three-dimensional (3D) section of reciprocal space that covers the region of interest. Consequently, the q_x-q_y plane at $q_z = 0$ was extracted by gridding the data onto a cuboidal mesh. This process leads to the typical REXS patterns shown in the main text. Subsequently, different photon energies around the Co and Cu L_3 edges were used for obtaining REXS patterns in the same reciprocal space regions, from which the REXS spectra were acquired. Note that a REXS spectrum is not the same as a XAS spectrum. The spectra shown in Figs. 2(e) and 2(f) in the main text are REXS spectra.

S3. LOOK-UP TABLE FOR DETERMINATION OF HELICITY ANGLE

For the following discussion, we treat skyrmions as 2D objects with non-zero topological winding number [2]. The full description of a 2D skyrmion can be represented using the local magnetization unit vector in spherical coordinates: $\mathbf{m}(\rho, \Psi) = (\sin\Theta\cos\Phi,\ \sin\Theta\sin\Phi,\ \lambda\cos\Theta)$, where (ρ, Ψ) are the real-space polar coordinates [2]. Such an axially symmetric expression can be understood by looking at the two structural parts. First, $\Theta = \theta(\rho)$ describes the radial profile, i.e., the out-of-plane magnetization component m_z that evolves from the center to the boundary of the skyrmion. $\lambda = \pm 1$ is the polarity, and we define $\lambda = +1$ corresponding to the core magnetization being parallel to $+z$, and vice versa, as shown in Figs. S3 and S4. Second, $\Phi = \Psi + \chi$ is the in-plane (m_x-m_y) configuration, where χ is the helicity angle [2]. Note that the chirality is defined as $\mathcal{C} = \lambda\mathcal{H}$, where the helicity $\mathcal{H} = \mathrm{sgn}(\chi)$.

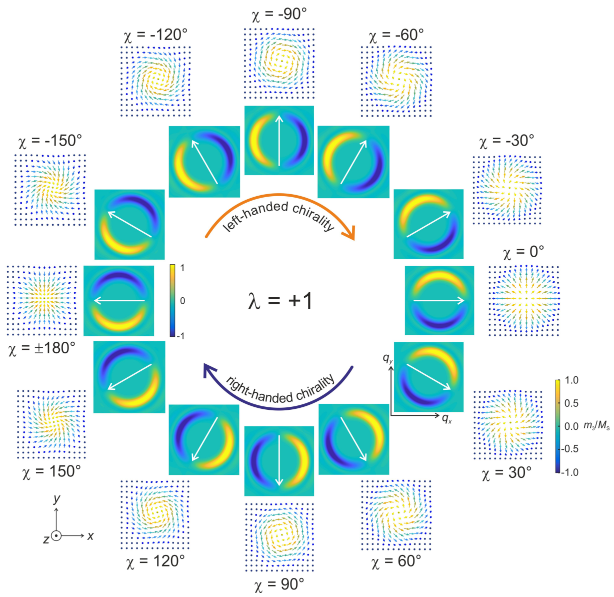

FIG. S3. Look-up table for the experimental determination of the skyrmion helicity angle χ for a system with positive polarity ($\lambda = +1$) using CD-REXS.

It is worth emphasizing the fundamental differences between the definitions of *chirality* and *helicity* of a

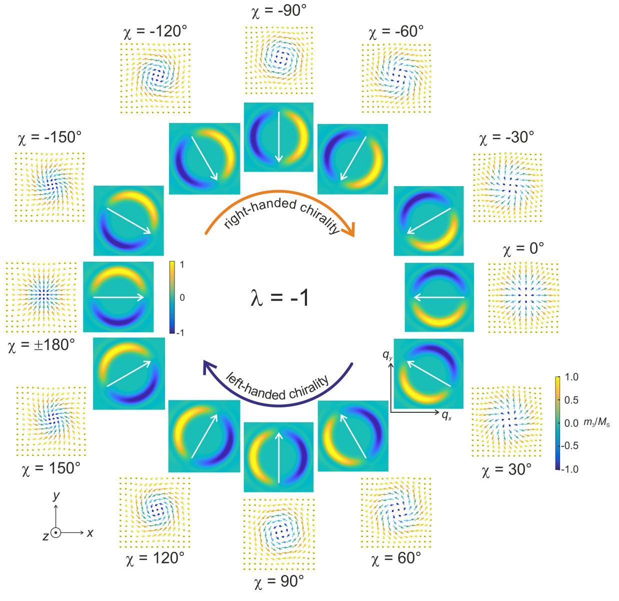

FIG. S4. Look-up table for the experimental determination of the skyrmion helicity angle χ for a $\lambda = -1$ system using CD-REXS.

skyrmion. The helicity $\mathcal{H}$ (not the helicity angle χ!) of a skyrmion can be understood as the rotation sense of the swirl in a particular projection plane, e.g., when looking down on a skyrmion in x-y-plane along the $-z$ direction, as shown in Figs. S3 and S4. Therefore, $\mathcal{H} = \mathrm{sgn}(\chi)$ can only acquire the values ± 1. Here we define $\mathcal{H} = 1$ (-1) to correspond to the counterclockwise (clockwise) rotation sense. On the other hand, the chirality of a skyrmion written as $\mathcal{C} = \lambda\mathcal{H}$ describes the handedness of the swirl, which can take the values ± 1. Thus, by flipping the magnetic field, the chirality is conserved, leading to both the polarity λ and helicity $\mathcal{H}$ reversed. In our case, the applied field $\mathbf{B} \parallel -z$, leading to $\lambda = 1$. Therefore, in the main text, we dropped λ in Eq. (1).

For the purpose of this work, we define the circular dichroism signal (the CD-REXS signal) as the difference in diffraction intensity for the same skyrmion peak at the same geometrical condition, obtained using left- and right-circularly polarized soft x-rays. Using CD-REXS, the helicity angle χ of a 2D skyrmion can be unambiguously determined [3–6]. REXS can be visualized as an elastic two-step process, in which a photon is emitted from the intermediate state after photon absorption. At the soft x-ray Cu $L_{2,3}$ edge, the process is dominated by electric-dipole transitions [7, 8]. The CD-REXS signal, as measured by the intensity difference of a magnetic peak using incident left- and right-circularly polarized x-rays, can be directly related to all three components of the magnetization vector [4, 8, 9]

$$I(\mathbf{q}) = 2|F_1|^2\,\mathrm{Im}\{[\mathbf{k}_s \cdot \mathbf{M}^*(\mathbf{q})](\mathbf{k}_s \times \mathbf{k}_i) \cdot \mathbf{M}(\mathbf{q})\}\ , \quad (1)$$

where F_1 corresponds to the photon energy dependent scattering factor, $\mathbf{k}_i$ and $\mathbf{k}_s$ are the incident and scattered x-ray wavevectors, $\mathbf{q}$ is the momentum transfer related to the reciprocal-space vector of the magnetic lattice, and $\mathbf{M}(\mathbf{q})$ are the Fourier components at $\mathbf{q}$. A 2D skyrmion lattice leads to six magnetic peaks. On the other hand, disordered 2D skyrmion lattices give rise to ring-like patterns in reciprocal space. Exposing a 2D skyrmion structure to Eq. (S1), and carrying out numerical simulations, yields a dichroic pattern in reciprocal space. These look-up tables are shown in Figs. S3 and S4.

For a well-ordered skyrmion lattice, the six skyrmion peaks have varying circular dichroism amplitudes, and can be separated into two opposite halves with positive and negative dichroism. The dichroism extinction vector is the oriented dividing line of the two halves (yellow and blue color on the left and right, respectively). Importantly, as can be verified from Figs. S3 and S4, the angle of the extinction direction directly corresponds to the helicity angle χ. In other words, while χ evolves from $0°$ to $360°$, the extinction direction rotates by the same angle.

Note that the angle of the extinction direction only depends on χ, and is independent of the other parameters. For example, for fixed χ, if the skyrmion lattice is rotated azimuthally (e.g., due to a sample rotation about the momentum transfer), the azimuthal angle of the six-peak pattern will rotate accordingly, however, the angle between the extinction direction and the x-axis will remain the same. This is due to the fact that while the lattice is rotated, the magnetic structure of each individual skyrmion is invariant for azimuthal rotation. If one constructs a skyrmion system composed of differently (in-plane) oriented lattices, all leading to different peak positions, we find the peaks to degenerate into a dichroic ring as shown in Figs. S3 and S4. This system represents the case of disordered skyrmions, each with the same χ. This dichroic ring pattern is therefore representative of the spin motif, and can also be used to study many-skyrmion systems that lack long-range order, such as multilayered thin films. It is then also clear that the extinction direction does not depend on the overall orientation of the sample in the setup.

The CD-REXS principle is summarized by the look-up tables shown in Figs. S3 and S4, and can be analytically written as [3–6]

$$I_{\mathrm{CD}}(\Psi) = \lambda Y \sin(\Psi + \chi)\ , \quad (2)$$

where $Y = 4|F_1|^2\pi^2k^2M_S^2\sin\theta$ is independent of Ψ, and $k = |\mathbf{k}_i| = |\mathbf{k}_s|$, M_S is the saturation magnetization of the material, and θ is the diffraction angle. λ strictly relates to the magnetic field direction $\mathbf{B}$, i.e., $\lambda = -\mathrm{sgn}(\mathbf{B})$ [2]. Therefore, in a CD-REXS measurement, once the field direction is chosen, λ is determined. In our case, $\lambda = 1$ throughout all measurements.

S4. MICROMAGNETIC SIMULATIONS

The micromagnetic simulations were carried out using `mumax`^{3} [10]. A system with an area measuring $240 \times 240\,\mathrm{nm}^2$ was simulated. The magnetic multilayers consist of four 1.5-nm-thick magnetic layers, separated by 4.5-nm-thick non-magnetic spacers. The thickness of the chiral magnet is 361.5 nm, and a 3-nm-thick non-magnetic spacer layer is inserted between the magnetic multilayer and the chiral magnet. The cell size is $3 \times 3 \times 1.5\,\mathrm{nm}^3$. Periodic boundary conditions are applied along the x and y directions.

In the simulation, the following parameters are employed for the magnetic multilayer: exchange stiffness $A = 1 \times 10^{-11}\,\mathrm{J/m}$, saturation magnetization $M_s = 0.95 \times 10^6\,\mathrm{A/m}$, perpendicular magnetic anisotropy $K_u = 5.5 \times 10^5\,\mathrm{J/m^3}$, and the interfacial type Dzyaloshinskii-Moriya (DM) constant $D_i = 1 \times 10^{-3}\,\mathrm{J/m^2}$. The following parameters are employed for the chiral magnet: exchange stiffness $A = 3.5467 \times 10^{-13}\,\mathrm{J/m}$, saturation magnetization $M_s = 104 \times 10^3\,\mathrm{A/m}$, and the bulk Dzyaloshinskii-Moriya constant $D = 7.457 \times 10^{-5}\,\mathrm{J/m^2}$. The Dzyaloshinskii-Moriya constant is set to be of interfacial type for the top 10 layers of the chiral magnet in order to capture the experimental observation [5].

* These authors contributed equally to the work.
† Corresponding authors.
E-mail: shilei.zhang@shanghaitech.edu.cn or thorsten.hesjedal@physics.ox.ac.uk

References

[1] K. Ran, Y. Liu, Y. Guang, D. M. Burn, G. van der Laan, T. Hesjedal, H. Du, G. Yu, and S. Zhang, Creation of a chiral bobber lattice in helimagnet-multilayer heterostructures, Phys. Rev. Lett. **126**, 017204 (2021).

[2] N. Nagaosa and Y. Tokura, Topological properties and dynamics of magnetic skyrmions, Nat. Nanotechnol. **8**, 899 (2013).

[3] S. L. Zhang, G. van der Laan, and T. Hesjedal, Direct experimental determination of spiral spin structures via the dichroism extinction effect in resonant elastic soft x-ray scattering, Phys. Rev. B **96**, 094401 (2017).

[4] S. L. Zhang, G. van der Laan, W. W. Wang, A. A. Haghighirad, and T. Hesjedal, Direct observation of twisted surface skyrmions in bulk crystals, Phys. Rev. Lett. **120**, 227202 (2018).

[5] S. L. Zhang, G. van der Laan, J. Müller, L. Heinen, M. Garst, A. Bauer, H. Berger, C. Pfleiderer, and T. Hesjedal, Reciprocal space tomography of 3D skyrmion lattice order in a chiral magnet, Proc. Natl. Acad. Sci. (U.S.A.) **115**, 6386 (2018).

[6] W. Li, I. Bykova, S. L. Zhang, G. Yu, R. Tomasello, M. Carpentieri, Y. Liu, Y. Guang, J. Gräfe, M. Weigand, D. B. Burn, G. van der Laan, T. Hesjedal, Z. Yan, J. Feng, C. Wan, J. Wei, X. Wang, X. Zhang, H. Xu, C. Guo, H. Wei, G. Finocchio, X. Han, and G. Schütz, Anatomy of skyrmionic textures in magnetic multilayers, Adv. Mater. **31**, 1807683 (2019).

[7] S. L. Zhang, A. Bauer, H. Berger, C. Pfleiderer, G. van der Laan, and T. Hesjedal, Resonant elastic x-ray scattering from the skyrmion lattice in Cu_2OSeO_3, Phys. Rev. B **93**, 214420 (2016).

[8] G. van der Laan, Soft x-ray resonant magnetic scattering of magnetic nanostructures, C. R. Physique **9**, 570 (2008).

[9] S. L. Zhang, G. van der Laan, and T. Hesjedal, Direct experimental determination of the topological winding number of skyrmions in Cu_2OSeO_3, Nat. Commun. **8**, 14619 (2017).

[10] A. Vansteenkiste, J. Leliaert, M. Dvornik, M. Helsen, F. Garcia-Sanchez, and B. Van Waeyenberge, The design and verification of MuMax3, AIP Adv. **4**, 107133 (2014)
